# Understanding the medical challenges for the diagnosis and treatment of bilateral pitting oedema in children: a qualitative study

**DOI:** 10.1371/journal.pgph.0004125

**Published:** 2025-03-18

**Authors:** Giulia Scarpa, Joseph Sagara, Christopher Mambula, Marie-Hortense Koudika, Fabrizio Loddo, Emelie Yonally-Phillips, Manal Shamseldin, Métrey H. Tiv, Emily Lynch, Nana Nimbu, Aimé Lulebo Mampasi, Nono Mvuama Mazangama, Akory Ag Iknane, Jihane Ben-Farhat

**Affiliations:** 1 Epicentre/ Médecins Sans Frontières, Paris, France; 2 University of Leeds, School of Environment, School of Nutrition, Leeds, United Kingdom; 3 Médecins Sans Frontières Paris (OCP), Paris, France; 4 Save the Children International, London, United Kingdom; 5 Ecole de Santé Publique de Kinshasa, République Démocratique du Congo (Kinshasa School of Public Health), University of Kinshasa, Kinshasa, Democratic Republic of the Congo; 6 Ministry of Health, Koutiala, Mali; Wageningen University & Research, NETHERLANDS, KINGDOM OF THE

## Abstract

Severely malnourished patients can present with bilateral pitting oedema, which is a common sign of Kwashiorkor. However, bilateral pitting oedema can also be an expression of other pathologies. In Mali and DRC, the number of children presenting with bilateral pitting oedema at MSF (Médecins Sans Frontiers/Doctors Without Borders) hospitals are up to 30% (Mali) and 49% (DRC) higher than in other countries, however, the reasons underlying this trend are unknown. Through this qualitative study, we aimed to explore the perspectives and lived experiences of health professionals on the diagnosis and management of children with bilateral pitting oedema. Using a participatory approach, we conducted 21 in-depth interviews, and 2 focus groups with health professionals at MSF health facilities who had worked in the settings of Koutiala (Mali) and Rutshuru (DRC) for at least 6 months. The understanding of the bilateral pitting oedema phenomenon is complex. Health workers described clinical obstacles to reducing mortality, including: i) difficulties making the diagnosis due to a lack of specialized staff and insufficient resources, ii) challenges treating complications that may arise due to the complexity of the diseases associated with bilateral pitting oedema, and iii) lack of scientific evidence in the literature explaining the physiopathology of bilateral pitting oedema. Study participants shared several key recommendations for reducing mortality among children presenting with bilateral pitting oedema, including prevention of bilateral pitting oedema at the community level, standardization of the diagnostic process, strengthening of medical training, and better collaboration both within the medical teams and between teams and the children’s families.

## Introduction

Hunger and undernutrition remain a major public health concern worldwide, affecting 8.9% of the global population [1]. Kwashiorkor is a severe acute form of malnutrition affecting young children, which is characterized by bilateral pitting oedema [2,3]. There are different theories about the etiology of kwashiorkor, the cause of which remains uncertain. However, it is certain that the prevalence of kwashiorkor is increased in certain parts of the world, including the Democratic Republic of Congo, Mali and Malawi [4]. Oedema is a clinical condition characterized by an increase in volume of interstitial fluid and swelling of the associated tissue. Bilateral pitting oedema involves legs and/or ankles. It can be localized or generalized, with differing degrees of severity (from the lowest degree with +, to the medium ++ until the highest degree +++) [2].

Bilateral pitting oedema may also be indicative of other pathologies outside of Kwashiorkor [5,6]. There are different causes for the development of these oedemas, such as venous stasis of systemic origin during congestive heart failure (or cardiogenic oedema), or as a sequalae of venous thrombosis, or renal causes, such as nephritis and nephrotic syndrome. Differentiating between nutritional oedema and oedema of another origin remains difficult with likely consequences for treatment and patient health outcomes [7].

A prospective cohort hospital-based study was conducted by Epicentre/MSF among 1611 children aged 6-59 months with bilateral pitting oedema in 2017 in DRC and Mali [4]. A total of 1614 children with oedema were admitted to the two centres over the course of the study. The refusal rate observed was very low, with only two refusals in Koutiala and one refusal in Rutshuru. As a result, 1611 children aged between 6 and 59 months were included in the study: 488 in Rutshuru and 1123 in Koutiala. At the two study sites, around half of the children enrolled with bilateral pitting oedema (54% in Rutshuru, DRC, and 51% in Koutiala, Mali) had concurrent acute malnutrition (moderate or severe) according to middle-upper arm circumference. The percentages were notably higher using the weight/height ratio criteria (87% in Rutshuru, 71% in Koutiala). The majority of the children were admitted with a medium or high degree of oedema in Koutiala (Mali) and with a low or high degree of oedema in Rutshuru (DRC). Also, most children were aged 12 months or more (97% and 98% in Rutshuru and Koutiala respectively). In Rutshuru, half of the children in the cohort were weaned and eating diverse foods; in Koutiala, the majority of the children were weaned, however only a few were eating diverse foods. The diets of the children in the study lacked protein intake. In Rutshuru, more than half ate a meal containing protein-rich foods a maximum of once a week; in Koutiala, half received no protein-rich food. Their diets were also lacking in vegetable and fruit consumption; a little over half the children in Koutiala and half the children in Rutshuru only ate vegetables or fruit 1-2 times a week. Results also highlighted differences in clinical and biological characteristics among children with bilateral pitting oedema, including its resorption. Half of the children were admitted with hypotension and half of them were anaemic.

During the Ben-Farhat study [4], health workers expressed different opinions about the causes and treatment of bilateral pitting oedema, including Kwashiorkor [8].

To better understand the challenges in diagnosing and treating children presenting with bilateral pitting oedema, we conducted a qualitative study, engaging clinicians, nurses and other health care staff at the local and international levels. This work aimed to strengthen the diagnostic and treatment process and reduce the high mortality rate among those patients.

## Methods

### Study design

In this study, we used participatory-action research (PAR), which is a systematic approach to discuss, and address participants’ needs, and guide future actions [9,10]. As a participatory approach, PAR considers participants to be equal contributors to the research process [11]. In this study, we also used a decolonising approach [12]: we consulted with participants to elicit the methods most suitable for exploring medical needs in the field, and, using these methods, investigated recommendations to improve the diagnosis and treatment of bilateral pitting oedema among local clinicians, nurses and assistant nurses to make sure contextually-relevant and acceptable actions could be taken.

The conception of this study and the aims and objectives emerged from those working in the field. Also, the framework used for the analysis was adapted with the contributions of the participants, and all the recommendations for next steps to improve processes and outcomes also reflect the insights of the participants. Some of the participants decided to engage in the writing of the manuscript and provided written feedback to the draft submitted to the journal, becoming part of the co-authors group.

To answer to our research questions, we used in-depth interviews (IDIs and focus group discussions (FGDs), which were critical to explore the lived experiences of health workers. These methods also helped to define the next steps to be taken at the local level among the communities, and within MSF supported health facilities to reduce mortality among children presenting with bilateral pitting oedema.

### Conceptual framework

**Fig 1:**
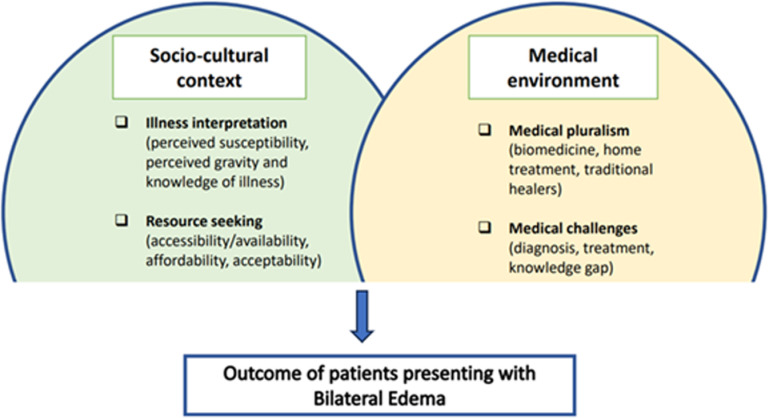
Conceptual framework of the study adapted from the PASS-model.

As a conceptual framework, we used an adapted version of the PASS-model (from PASS International organization, with whom the model was built), which was contextualized in collaboration with the research participants (Fig 1). This framework is widely used in health research, especially in low-income settings [13]. It is divided in two main areas: the “socio-cultural context” and the “medical environment”. Concerning the “socio-cultural context”, we focused primarily on the *illness interpretation,* in which health workers described bilateral pitting oedema from a medical perspective, and *resource seeking,* with evaluation of MSF health services’ accessibility. The “medical environment”, which we explored more deeply, included the sub-categories of *medical pluralism,* with the analysis of the use of traditional treatments and biomedicine to cure the disease*,* and “medical challenges” (a section added for the purposes of this study), in which we assessed the difficulties in diagnoses and treatment.

### Study settings

**Fig 2:**
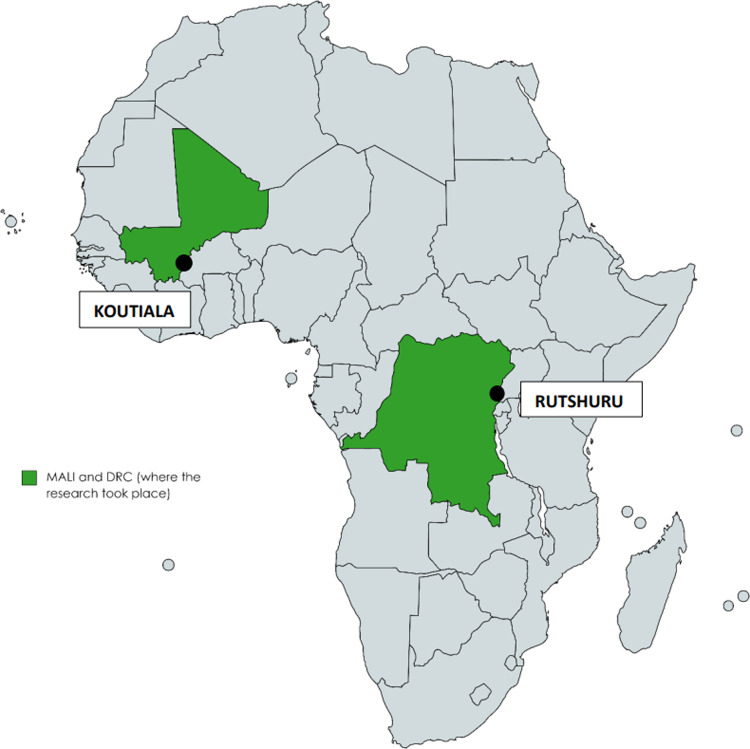
Settings where the research took place.

This research was conducted in Koutiala (Mali) and Rutshuru (DRC) (Fig 2), where approximately 16 to 49% of children presented with bilateral pitting oedema at MSF hospital admission [4].

#### Koutiala, Mali.

Koutiala is a city in Sikasso region, Mali. A severe humanitarian crisis has been ongoing across the country since 2018 due to conflict, extreme climatic events, displacement, and high food insecurity, leading to malnutrition. The most affected have been children and women, with high level of poverty across the country [14]. According to UNICEF [15], 4.1 million people were in need of humanitarian assistance due to lack of access to health services, clean water, and schools. Climate change has contributed to the increase of malnutrition, due to erratic rainfall, poor agriculture production, desertification and increment of crop pests [15]. The national prevalence of acute malnutrition is 9%, and the prevalence of stunting in children under 5 years is 27% according to USAID in 2022 [16].

MSF has worked in Koutiala since 2009 in collaboration with the Malian Ministry of Health (MoH), to improve prevention and treatment services, particularly for young children. MSF supports both in-patient and out-patient paediatric malnutrition care services at a reference hospital, and several health facilities.

#### Rutshuru, DRC.

Rutshuru is in the North Kivu province in the east of the Democratic Republic of Congo. There are two rainy seasons, in which malaria and malnutrition prevalence are higher. The first one is from March to May and the second from September to December, although with climatic change the seasons are becoming more unpredictable [17]. North Kivu is affected by conflict, especially in the eastern regions, along the borders with Rwanda and Uganda. As a consequence, episodes of violence, displacement, and difficulty in accessing health facilities have increased, with a negative effect on the local communities, especially women and young children [18]

In Rutshuru, MSF provides paediatric services and malnutrition care at three hospitals and five health centres since 2005. Based on USAID data (2022), 12% of children under age 5 were affected by severe acute malnutrition (SAM), and 42% by stunting. Malaria, respiratory infections and diarrhoea are also leading causes of morbidity and mortality in young children in this setting [19].

### Participant sampling

Purposive sampling was used to ensure as many health professional perspectives as possible in relation to the study’s objectives, including nurses, nurse-assistants, and physicians at the international, national, and headquarter levels (medical and nursing staff) in Mali and DRC. To be included in the study, all participants were required to have worked in Mali and/or DRC MSF health facilities for a minimum of 6 months to get enough experience with children presenting with bilateral pitting oedema in Koutiala and/or Rutshuru. Additional participants who met the stated inclusion criteria were identified and recruited through snowball sampling. Two FGDs were conducted in total, one comprised of Malian and Congolese nurses and nurse-assistants, and one comprised of Malian and Congolese physicians.

### Data collection

Fieldwork was conducted from 3 June 2021 to 25 March 2022 in DRC, and from 3 January 2022 to 25 March 2022 in Mali. Local, expat and headquarter personnel participated in the IDIs and FGDs. IDIs and FGDs included semi-structured and unstructured questions (S1 Text and S2 Text). Prior to the data collection, the questions were piloted among MSF staff to assess for clarity. Written consent was obtained before conducting the interviews.

Data collection was conducted in French and English to accommodate the participants’ needs. Secure online platforms were used to conduct IDIs and FGDs, and field researchers transcribed the information verbatim. Data collection was stopped when saturation was reached (no new information added). The average length of time was 50 minutes for in-depth interviews and 95 minutes for focus group discussions.

### Data analysis

Thematic analysis, including latent and semantic approaches, was used to analyse the information collected. We followed the six standard steps for this type of analysis: data familiarization, coding, generating themes, reviewing themes, defining and naming themes, and writing findings [20]. The information was deductively and inductively coded using NVivo 12 Plus. The PASS-model was used to create the pre-defined categories, while emergent sub-themes were inductively coded. Data was also triangulated by assessing themes and information available in both IDIs and FGDs.

### Ethical considerations

Ethics approval was received for this study from the Comité National d’Ethique de la Santé et les Sciences de la Vie in Mali (2022), Comité National d’Ethique de la Santé in DRC (2021), and MSF ERB (2021).

#### Reflexivity statement.

The authors of this article have different backgrounds, but all have experience in public health research projects. Many authors have a medical/nursing background or global health research expertise. Most of them have clinical or research experience in non-governmental organizations (Medecins Sans Frontiers, Save the Children). However, the place of origin and work vary (Europe, Sub-Saharan Africa, North America). Five co-authors are from DRC, and one is from Mali.

Our experiences in participatory research and decolonial methods influenced the design of this research as well as the type of engagement with the participants on the ground.

Some of the participants worked closely in DRC or Mali with some co-authors, but not with the first or last author of the paper, who were primarily involved in the data collection and analysis. Therefore, the potential for bias is possible, but limited. For the interpretation/discussion of some specific results, for example needs of health workers for improving the diagnosis of bilateral pitting oedema, co-authors may have reflected their own experience informing the final recommendations. However, due to the participatory nature of this research and the close relationship between participants and researchers, any recommendations and final results have been discussed and agreed with the participants to make sure they are context-relevant and in line with the current medical practice.

## Results

We conducted 21 in-depth interviews, and 2 focus group discussions for this study (Table 1). The acceptance rate to participate in the research was 100%. The majority of participants (23/25 = 92%) had over 5 years work experience in nursing or medicine. Three participants had worked in both Rutshuru and Koutiala settings with local and expat contracts.

**Table 1:** Participant characteristics for this study.

Participants characteristics	Total per category	Number of participants
**Role**		
*Local staff*	n=11	
Physicians		4
Nurses		4
Nurse and nutrition assistants		3
*Delocalized expatriate (international)*	n=8	
Physicians		4
Nurses		4
*Headquarters*	n=6	
Experts in nutrition (physicians and nutritionists)		6
**Sex**		
Female		9
Male		16

All participants reported that to prevent and cure nutritional and non-nutritional bilateral pitting oedema it was necessary to investigate the socio-cultural, environmental and economic factors, as well as the medical practice, aligning with the use of the adapted PASS framework which we use to present our findings below.

### Socio-cultural context

#### Illness interpretation.

Health workers described their perspectives regarding the causes of bilateral pitting oedema in the community. All participants, when talking about ‘bilateral pitting oedema’ first mentioned ‘malnutrition’ or ‘nutritional oedema’ more than any other diseases. Participants reported that Kwashiorkor patients were particularly at risk of death. While the underlying causes of this type of malnutrition are still unknown, health workers reported that Kwashiorkor was probably the result of an imbalanced diet, low in proteins, fats and vitamins.

Health staff who had worked in both Mali and DRC highlighted some differences in the two countries, namely that in Mali the patients who accessed MSF facilities were more familiar with nutritional oedema, and that health services were more reachable than in DRC.

Additionally, health workers in Mali and DRC described specific periods of the year during which greater numbers of patients with bilateral pitting oedema accessed MSF health facilities, which they linked to the agricultural period and season prior to harvesting, when food insecurity was higher. Additionally, it was frequently mentioned that malnutrition and bilateral pitting oedema cases were likely to increase during malaria and measles’ peak occurring yearly.

The majority of the health professionals reported that bilateral pitting oedema was a serious issue in both locations with the potential for severe health outcomes for their patients. However, one participant noted that the perceived severity of nutritional oedema among health workers could be overshadowed by the presence of other diseases considered to have higher risk of mortality.

We manage malnutrition, [but] we don’t take it very seriously (Male participant, IDI)

#### Resource seeking.

High transportation costs, inconvenient geographical locations, inaccessibility of roads in both settings, and insecurity due to armed clashes in DRC were among the barriers listed by the participants to bringing a child to MSF health facilities. Local health workers living in Rutshuru and Koutiala reported that many of their patients described accessing health centres as more expensive than being treated by traditional healers. They added that families accessing their health facilities were worried that MSF hospitals would ask for money to treat the child. According to them, many families, who were treated at MSF hospitals were not aware of the free treatment in MSF health centres, especially in Rutshuru. In fact, in Rutshuru the in-patient paediatric department was run by Ministry of Health’s staff, and there was previously a fee to pay at the admission. According to participants, there was higher awareness regarding MSF in Koutiala:

[...] people are well aware of MSF’s activities and they know that when you go to the hospital, you have access to free care […] we’ll say for certain criteria only (you are referred to MSF centres), and when you arrive at the hospital, there’s a sorting process, and according to the sorting process, you’re referred there (to MSF), but I also know that there are many people who go to the hospital, hoping to be referred to MSF (Male participant, IDI).

Local health workers reported that at the MSF hospitals in Rutshuru and Koutiala children often arrived very sick, reducing chances of successful treatment. They stated that caregivers often reported not feeling comfortable in many health settings because of previous experiences of mistreatment, which contributed to decisions to delay seeking care at the hospital until the child was very sick:

So they don’t believe in what happens at the hospital, often people say that when you arrive at the hospital, they don’t treat you properly, so people still prefer to leave, to be treated by the traditional therapist (Male participant, IDI).

Also, local health workers reported that both Koutiala and Rutshuru have a patriarchal society, in which the father holds the responsibility of decision-maker. However, they noted that the mother was usually the one accompanying the sick child to the health facilities, and generally looking after the child when sick. Thus, participants thought that more involvement of the entire family in the childcare would have a positive impact on child’s health:

And if we manage to involve the parents, which is not only the mothers, especially in our context, here in Mali, decision-making on women is a bit complicated. It’s only the men. But if we manage to approach these men, to discuss with them, to make them understand, to involve them in the care, I think that can help us a lot (Female participant, IDI).

### Medical environment

#### Medical pluralism.

Participants from both sites explained that, when the child was sick, caregivers could access traditional remedies, which included home treatment and the use of traditional healers, and medical treatment, which included the use of pharmacies and health facilities.

During the focus groups, health workers from Mali and DRC agreed that that taking children to traditional healers and administering traditional drugs first could make their recovery more complicated, given that the dosages administered during traditional treatments were unknown by the medical staff. They also explained that children with bilateral pitting oedema were very vulnerable to organ damage, and if the correct diagnosis of oedema was not made, traditional drugs could worsen the situation, leading to higher risk of death:

And then you see with traditional products, we don’t know the dosage. We don’t know the active ingredients of what we are giving. And most of the time, a child who has taken a lot of traditional products is difficult to be saved [...] And if it’s a malnourished child, liver and kidneys are already damaged. […] These children get a lot of poisoning (FGD).

#### Medical challenges.

##### a.Diagnosis challenges

The participants noted that there were challenges all along the pathway of care, beginning when the child arrived at the hospital. First, they reported that during the triage process, the nurse or nutrition assistant usually took the anthropometric measurements (weight, height, middle upper arm circumference) and assessed the bilateral pitting oedema to check the nutritional status. A physician only re-assessed the patient, including their oedema status, if the nurse or nutrition assistant identified any out-of-range parameters. This was perceived by the participants as a limitation in the diagnosis process. No major differences were found in terms of clinical diagnosis’ practice between the staff working at Rutshuru and Koutiala health facilities.

Many of the participants from both sites reported that the differential diagnosis of nutritional oedema was quite challenging:

But we had several discussions even among, let’s say, experts, for example saying, if you have oedema on the tibial area but not on the feet, is that oedema good enough to diagnose Kwashiorkor? So I think it’s not an easy diagnosis to make. It’s something that we give for granted because we said, come on, you do your MUAC, you push on the feet and that’s it. But it’s not that simple. Plus, it’s true that you can have differential diagnosis. […] In an ideal world, Kwashiorkor is diagnosed when you have ruled out other possible causes of oedema. But in our context, it’s first, very difficult, second, people are just so used to say ‘there is malnutrition, there is oedema, it’s Kwashiorkor’ (Male participant, IDI).

Moreover, one participant explained that the misdiagnosis of bilateral pitting oedema could happen easily given that the initial patient screening (triage) was often undertaken by an assistant nutritionist. However, other participants argued that nutrition assistants, who were not doctors or nurses, were well-trained before making differential diagnosis, and, also, well-supported by senior staff.

Participants working in Koutiala and Rutshuru reported that all patients with bilateral pitting oedema were first treated as having nutritional oedema, unless there were clear signs of a different pathology. The children were usually given nutritional treatment, and then the situation would have been monitored over a timespan of a few days. If the child did not improve, a new diagnosis was made, and new investigations were undertaken. In most of the cases, participants reported that making a diagnosis was possible with the available tests/instruments, which included urine sticks, ultrasound machine, and rapid screening test for tuberculosis. However, staff working in Rutshuru agreed that there were challenges in getting the laboratory tests to detect renal-related diseases, while in Koutiala this was not identified as a problem.

The health workers agreed in the focus groups that there were some important issues if an incorrect diagnosis of kwashiorkor in the setting of bilateral pitting oedema was made, including: i) loss of time to treat the real illness, which could be fatal in the case of cardiac problems; ii) the patient could be overtreated, for example getting antibiotics when unnecessary, potentially leading to antibiotic-resistance; iii) reduced time to refer the patient to another clinic/hospital where he/she could be treated; iv) not well-supported with palliative care when needed, by stopping unnecessary treatment. Moreover, they reported that many diseases presenting with bilateral pitting oedema were identified as treatable, for example severe anaemia or nephrotic syndrome, if correctly diagnosed. According to the health workers at headquarter level, while cardiac conditions were unlikely to be cured, making the diagnosis was necessary for being honest with the family, setting expectations, and providing support during the process.

##### b. Treatment challenges

In the focus groups, participants agreed that treating the diseases associated with bilateral pitting oedema could be challenging. The main problem identified was that patients were often too sick to have a good prognosis, especially in the case of nutritional oedema. Health workers explained different factors that could worsen the outcome of patients presenting with nutritional oedema which were themselves difficult to treat, including: i) sepsis due to skin lesions; ii) metabolic disorders, often leading to hypoglycaemia; iii) other associated pathologies, for example renal or cardiac; iv) imbalance of water and electrolytes, particularly when patients present with diarrhoea; v) complications, such as gastric dilatation.

Some discrepancies also arose in health worker descriptions of directions for ongoing treatment post-discharge. For example, while health workers stated that in the case of nutritional oedema, the caregiver should be continuing the nutritional treatment at home with Ready-to-Use Therapeutic Food (RUTF), whether supplemental foods were also allowed was contested. Some health workers especially in Rutshuru, indicated that it could be given in addition to other food, while others stated that, per Malian guidelines, only RUTF was allowed without any supplemental foods.

#### Health worker recommendations.

During the FGDs and IDIs, health workers discussed recommendations to improve the outcome of children presenting with bilateral pitting oedema (Table 2). Strengthening prevention and collaboration, standardizing the steps of the diagnosis process, hiring additional and specialized medical staff, and investing in research were found to be essential to improve outcomes of patients presenting with bilateral pitting oedema.

**Table 2:** Recommendations agreed by the health workers during the study in mixed focus groups (local health workers from both countries) to be applicable for Mali and DRC context.

Areas to be improved	Practical recommendations	Expected outcome	Theme(s) in the PASS-model
Prevention	Having a physical presence among the community to create a two-way dialogue. Particular attention to communication with families when the child is hospitalized should also be prioritized. Implementation of radio channels and partnerships with other non-governmental organizations in rural areas and with community leaders Strengthening of education on complementary feeding, and IYCF (infant and young child feeding) in the community	Improve families’ understanding of the diseases linked to the bilateral oedema, its gravity and susceptibility, and incentivize early child referral	Illness interpretation, decision making, medical pluralism
Collaboration	Elimination of hierarchical barriers among the team (physicians, nurses, nurses’ assistants) Open dialogue and collaboration with caregivers Collaborations with traditional healers	Improve diagnosis and treatment of patients by better understanding the patient’s history, and better explaining post-discharge care to the families & incentivize early child referral	Medical challenges, decision making, illness interpretation
Standardization of diagnosis	Specialized training for triage staff, and ‘refresher sessions’ afterwards Standardized process of diagnosis i) rapid screening test for tuberculosis, ii) screening of urine, iii) detailed patient history to collect with the caregiver Appropriate lab testing capacity	Improve diagnosis of bilateral pitting oedema by using a standardized process across all countries and settings	Medical challenges, medical pluralism
Staff	Higher number of health workers for the hospitalized patients Psychological support to implement in the hospital	Improve treatment and care during the patients’ hospitalization, and support caregivers	Medical challenges, Illness interpretation
Research	Understanding the causes of death in patients presenting with bilateral pitting oedema Possibility to conduct autopsies	Improve knowledge, treatment and outcomes of patients presenting with bilateral pitting oedema	Medical challenges

During the FGDs, particular attention was paid to the strengthening of communication between medical staff and caregivers to improve prevention and health outcomes in patients:

The medical team is too focused on care, and often forgets about the mother. For me, we should put more emphasis on raising the mother’s awareness, explaining to her what illness her child has, and also explaining to her how this type of pathology can be avoided [...] if she sees another child who has the same symptoms, the same problems, so she can explain (to another mother) to be careful, to go to the hospital (Female participant, FGD)

Also, health workers agreed that additional and specialized staff is critical to properly monitor patients presenting with bilateral pitting oedema, particularly Kwashiorkor, whose condition can deteriorate very quickly if not constantly monitored:

Because for me, Kwashiorkor children need special attention, you know, in a care room, for me a Kwashiorkor child, you have to find a space, group them together, do a more special monitoring as if it was in intensive care even if they are not yet in the intensive care charts […]You cannot look after a Kwashiorkor child like any other child. A Kwashiorkor child can take the milk at 2pm, drinking everything, normally, then when you come back at 2.30pm, he can have a sudden cardiac arrest, like this (Male participant, FGD).

Participants working in Rutshuru agreed that more training would have helped to better diagnose patients presenting with bilateral pitting oedema. In Koutiala, most of the staff reported to have sufficient skills to make the diagnosis without needing further education.

Probably we should invest a bit more time to train at the different levels, from the Community health workers to the people that are doing the triage at the entrance of the child, that’s on the paediatrician, that is doing them MUAC, the weight for height and the assessing oedema. And sometimes when the child comes in with, the child has oedema, nobody is really questioning if it’s true or not. So you know in all the different steps of the diagnosis process and the assessment process, people should be properly trained (Female participant, IDI).

Finally, participants agreed that further research is needed to understand the physiopathology, improve the treatment, manage complications, and reduce child mortality in patients presenting with bilateral pitting oedema. They said they use the same treatment for marasmus, which is another type of non-oedematous malnutrition, though this may not be optimal for children with Kwashiorkor.

My feeling is that we kind of gave up trying to understand what Kwashiorkor is. So, it doesn’t sound challenging anymore, you know. There have been, I mean not much research, but many hypotheses […] and none of it was kind of confirmed. So difficult to understand because even children in the same family at different ages they come to the hospital, both with malnutrition, one with Kwashiorkor and one without. […] some children will have Kwashiorkor and you will never be able to understand why, you know (Female participant, IDI).

## Discussion

### What this research adds

To our knowledge, this is the first qualitative study investigating the socio-cultural and medical context of children presenting with bilateral pitting oedema whether of nutritional or non-nutritional origin, in Mali and the DRC from the perspective of health workers. Diagnosing bilateral pitting oedema has emerged as a critical step in medical practice, and the systematic collection and analysis of data on both Kwashiorkor-related and non-Kwashiorkor oedema remains a global challenge [21]. The report published in 2016 by the CMAM forum demonstrated that some countries have a relatively high proportion of SAM cases presenting as Kwashiorkor (for example, 32% in DRC). This highlights the heterogeneity of Kwashiorkor’s distribution within countries and regions, where it is endemic [8].

This research also builds on and provides a more contextual understanding of the quantitative findings from the previous study undertaken by Epicentre/MSF in 2017 [4], highlighting the complexity of the diagnosis of bilateral pitting oedema in children in such contexts.

Participants from Rutshuru and Koutiala reported that children presenting with bilateral pitting oedema (nutritional or not) usually start the nutritional treatment as per medical recommendation. From the quantitative study (4), we know that nutritional management at the therapeutic nutrition centre usually includes treatment with therapeutic milk (F75) followed by RUTF. Among the children recovered/stabilized, 97% of children in Rutshuru and 95% of children in Koutiala received these therapies. However, a small but significant proportion of children received only one of the two treatments, or neither. In Koutiala, 25 children received therapeutic milk (F75) without RUTF and 22 children received neither milk nor RUTF. These children had higher levels of proteinuria (50%) and hypertension (19%) on admission compared to those who received both milk F75 and RUTF (7% with proteinuria and 2% with hypertension). However, there was no difference in the presence or absence of skin lesions and apathy, two clinical features associated with Kwashiorkor. In both sites, 90% of children have been stabilized, 3% cured and 7% died. Fifty-three percent in Rutshuru and 61% in Koutiala were stabilized within 3-7 days of admission. The vast majority of children discharged cured or stabilized received a nutritional treatment based on F75 milk and RUTF at both sites.

This qualitative work was much needed as literature on the nutritional and non-nutritional differential diagnosis of oedema in children living in sub-Saharan African countries and the causes of death are limited. A recent study explored the key predictors of nutritional oedema among children [22]. Low maternal attendance to antenatal care services appointments and episodes of diarrhoea in the previous 2 weeks were strongly associated with the presence of bilateral pitting oedema. Other studies investigated characteristics of bilateral pitting oedema in renal and cardiac diseases [23–25]. However, there are no qualitative or mixed-methods components in these studies assessing why and how the diagnosis of nutritional and non-nutritional bilateral pitting oedema is done, and the treatment given. As noted in a recent article (2023) of ‘The Lancet’, ‘Kwashiorkor is often overlooked’ as published studies on this topic are very limited [21]. This work aims to begin to address this evidence gap on Kwashiorkor and pitting bilateral oedema research to improve the diagnostic process and reduce mortality.

### Main contributions of this research

#### Research design.

This study has been conceptually designed using participatory approaches, and a decolonising lens and methods. We provided a platform for health workers in various roles to come together and discuss an often-overlooked issue: the complexity of diagnosing and managing nutritional and non-nutritional oedema. This enabled the comparison of diverse perspectives and facilitated consensus on the direction that practice and research should take to improve the lives of children presenting with bilateral pitting oedema. The decolonising approach [26] allowed local health workers to raise their voice, and explain their needs in order to be considered at higher level (headquarter staff is, in fact, often based in high income countries), and their data used for action. Decolonising research, in our work, means empowering health staff based in the Global South, and balance the power between local clinicians and nurses, and headquarter workers.

#### Research content.

In this work, we summarised and critically appraised the recommendations of the interviewees to improve the understanding, diagnosis and treatment of bilateral pitting oedema, and to tailor on-the-ground actions to the community needs to reduce children mortality.

Health workers identified five areas of improvement at the community and medical level to ameliorate the health outcomes of children presenting with bilateral pitting oedema. First, *prevention* has been identified from all health workers as key element to reduce, early detect and treat bilateral pitting oedema, increasing the chance of patients’ survival. Working closely with community health workers and community leaders on prevention of malnutrition, including health and nutrition promotion (for example, on Infant and Young Child Feeding- IYCF), will help to: i) prevent malnutrition cases, ii) increase utilization of health facilities, iii) eliminate stigmatization about the bilateral pitting oedema among the most vulnerable [27], and iv) increase the utilization and access of patients to specialized medical centres and hospitals [28]. In parallel, as poverty is an underlying cause of malnutrition, all poverty-reducing interventions are needed to decrease the prevalence of malnutrition and related nutritional oedema over the long-term [29].

Second*, collaboration* between staff members in the MSF health facilities was found to be essential for the correct diagnosis and treatment of children presenting with bilateral pitting oedema. They highlighted challenges arising from the lack of brainstorming between junior and senior staff, lack of child deaths review, and medical assessment on admission conducted by less experienced health workers. Collaboration has the potential to have a positive effect on patient outcomes [30]. According to a systematic review [31], health workers should first work together to establish roles, tasks and responsibilities prior to caring for patients. More integrated collaboration models which are also patient-centred, and more specialized training for existing staff could reduce the tensions among the team in the best interest of the patient [32,33]. Also, a routine training schedule could help expatriate staff to learn procedures on site and contextualize medical practice in the field.

Third, *standardization of practices* was considered to be a critical element for the implementation of care in patients with bilateral pitting oedema in Mali and DRC. The participants emphasized that the entire health team should receive adequate training, have access to appropriate tests, and have clearly defined roles to improve the differential diagnosis in patients presenting with bilateral pitting oedema. Participants also highlighted the disagreement among health professionals working both in Mali and DRC about the use of feeding treatments at home (promoting RUTF along with home meals or RUTF alone), which varied according to the person in charge in the nutrition unit. According to the literature, the standardization of the medical practice would, in fact, reduce variations in clinical treatment, by improving the quality of care and decreasing potential errors [34].

Fourth, lack of *staff* and specialized health professionals in the hospital settings was found to be a major challenge during the diagnosis and care processes of patients with bilateral pitting oedema in both countries. The nurse to patients ratio was not ideal, according to the participants, as it exceed 5 patients per nurse. The low number of medical staff has been documented in literature, especially in middle and high-income countries [35]. According to a recent study, if the number of doctors, and especially nurses, is adequate—the ideal ratio in low to middle income countries is one nurse for four patients in medical and surgical units—many lives could be saved. In addition, this nurse to patients ration was shown to reduce the length of hospitalization, which has the additional benefit of decreasing the cost and resource burden for the hospitals [36]. Also, lack of psychological support for the family was identified as a barrier. Participants reported that often there were no available health workers specialized in psychology to help families in need. A life-threatening illness, which is the case of many patients presenting with bilateral pitting oedema, can cause high levels of stress, both psychologically and physically, for the patient and the family [37]. Psychological support for the families can help during such times and can have a beneficial impact on the patient as the family is often the patient’s primary emotional support. Psychological support for the family in this way also acts as a facilitator for the patient’s recovery as well as protecting the mother-child dyad, which is usually at risk in case of severe malnutrition. According to Babaei and Abolhasani’s study, only the family can strengthen the feeling of love and belonging during the time of sickness, and provide care which is culturally appropriate for his/her sick family member [38]. Intervention such as the psycho-stimulation of severely malnourished children is necessary to ensure better outcomes [39].

Fifth, lack of *research* about the causes of death in children presenting with bilateral pitting oedema, was considered to be an important limitation for advancing care and improving health outcomes. The participants reported that conducting autopsies could help in identifying causes of death to reinforce the differential diagnosis. Many middle and low-income countries lack nationally representative *causes of death* data [40–42]. Indirect methods are much less reliable than direct methods, which are critical to establish country priorities (31). Also, research on better diagnostic methods would help to disentangle the causes of bilateral pitting oedema in Kwashiorkor and non-Kwashiorkor cases [43], by improving patient management, and reducing the burden of child deaths.

During the interviews and FGDs, participants discussed about the causes of Kwashiorkor, for which the aetiology remains unclear [44]. Although more research is needed to better understand the physiopathology of nutritional oedema, this work highlights the need of investing in nutritional research and finding *ad hoc* treatments to reduce mortality for children presenting with bilateral pitting oedema. In practice and in the literature the treatment for Kwashiorkor and marasmus (malnutrition presenting with non-pitting oedema) is the same [45]. However, further research would be useful to better understand if the two different nutritional status’ require different treatments to improve the health outcomes.

This work together with the first quantitative component provide an idea of the trend of Kwashiorkor in Rutshuru and Koutiala, for which estimates were previously unclear, and often missing in the literature [21].

#### Further clinical recommendations.

Some additional clinical recommendations can be made by looking at our findings.

In terms of the diagnostic process, children presenting with bilateral pitting oedema and other signs of malnutrition (measured through the middle upper arm circumference or weight-for-height z score) should be immediately started on the nutrition treatment, however it is important to evaluate other possible co-morbidities to exclude a differential diagnosis. On the other hand, if the child does not have any other clinical signs of malnutrition rather than the pitting bilateral oedema, the nutritional status should still be closely evaluated as discussed by the participants. Also, the assessment of food security, weight loss, changes in diet pattern and other co-morbidities could indicate the presence of underlying severe micronutrient deficiency needing nutritional treatment [46,47].

In terms of patient management, cares should be tailored to the needs of the child [48]. Children presenting with bilateral oedema should possibly be placed in nearby nursing station and vital signs frequently monitored (every 3 hours) by the medical and nursing staff. The participants reported that children presenting with bilateral oedema can worsen unexpectedly and quickly. Good hygiene practices would also reduce the risk of nosocomial infections [49]. Health workers discussed the assessment of nutritional status and feeding practices during the hospitalization, noting that feeding and hydration should be assessed and ensured in a timely manner, and adequate feeding support measures should be put in practice if needed (e.g., use of nasogastric tube). Breastfeeding should always be supported, and skin-to-skin encouraged to prevent the risk of hypothermia and promote the psychosocial wellbeing of the mother-child dyad [47].

The impact of Kwashiorkor is significant, with a high mortality rate in young children [8]. Therefore, policymakers and researchers should engage more actively in addressing the causes of mortality related to Kwashiorkor and other bilateral pitting oedema pathologies to save the lives of many affected children worldwide.

### Strengths and limitations

There are some strengths to this study. First, the qualitative methodology helped to explore medical needs in the still relatively unknow field of bilateral pitting oedema. We were able to recruit health workers with different backgrounds and experiences to enrich the quality and rigor of this work. The qualitative approach gave voice to health workers in fragile settings, in which there is a high percentage of children presenting with pitting bilateral oedema. The enthusiasm and active participation of the health workers strengthen the study findings, identifying more tangible actions for daily practice. Participants reported to be keen in discussing more with other staff members in their health centres/hospitals about the challenges of diagnosis and treating children presenting with bilateral pitting oedema. They felt that this work was much needed and provided the opportunity to discuss and learn from each other.

However, there are some limitations in this study. All the interviews were conducted remotely, which can limit the researcher-participant rapport building, as well as limiting observations of body language and context. However, it should be noted that participants did not feel the online nature of the interview was a limitation, and many requested follow-up conversations to further share experiences and knowledge on bilateral pitting oedema. Additionally, while initially interviews with families of children presenting with bilateral pitting oedema were planned for this study, the Covid-19 pandemic and lockdown measures made this infeasible. As a result, data collected on interpretation of the illness, resource-seeking and decision making (constructs of the PASS model) are limited as they represent the experiences and perceptions of health professionals only, and do not include the perspectives of patient caregivers. Further research should include the perspectives of caregivers and sick children to complete the description of the socio-cultural context of the bilateral pitting oedema in Mali and DRC.

Also, the interviews and focus groups were conducted by a European researcher working for Epicentre/MSF, who did not know the health staff working in Rutshuru and Koutiala, and used a participatory and decolonising approach. However, the lived experience and background of the researcher could have biased the data collection and interpretation of the results.

Any interview that collects retrospective data is subject to limitations in recall. Some details about health workers’ experience in diagnosing and treating children with bilateral pitting oedema or discussing the socio-cultural context and barriers may be missed or misremembered. Social desirability bias is another possible limitation as the interviews were carried out by an MSF/Epicentre researcher with MSF personnel. However, data triangulation using different qualitative methods helped ensure quality and rigor.

Finally, the results of this study reflect the perspectives of health professionals in Rutshuru and Koutiala, therefore they cannot be generalizable. However, challenges about the diagnosis and treatment of nutritional and non-nutritional oedema may provide transferable informative for other hospitals and health centres in sub-Saharan African settings, and other vulnerable places where malnutrition is high.

## Supporting information

S1 Text Interview guide. (DOCX)

S2 Text Focus group guide. (DOCX)

S1 Checklist Inclusivity in Global Research questionnaire.
(DOCX)
