# Supplementary material for: Understanding the medical challenges for the diagnosis and treatment of bilateral pitting oedema in children: a qualitative study
Source: PLOS Glob Public Health. 2025 Mar 18;5(3):e0004125. doi: 10.1371/journal.pgph.0004125 (PMC11918406; doi:10.1371/journal.pgph.0004125)
Supplement: S1 Text — (DOCX) [file pgph.0004125.s001.docx]

S1 Text: Interview guide

This interview will not be used as an evaluation of your work. You are not being tested on your knowledge about oedema in children. We want only to know your opinion and perceptions about the process of diagnosing the causes of bilateral edema.

1. *Demographic information:*

How long have you been working for as a health care provider? How long have you worked for MSF? What is your current role? During your mission with MSF, what was your role? For how long did you work with children presenting bilateral edema?

2. *Access to care:* (Questions for local health workers only)

What do you think are the main problems encountered by families in accessing care? How do children with edema get to MSF facilities?

3. *Understanding and diagnosis of children with bilateral edema:*

What is bilateral edema for you (e.g. definition and clinical picture)? What do you think it is the understanding of edema in the community? What are the reasons or causes for edema in your opinion? Do you see different categories of patients with bilateral edema and how do you interpret each one? Which differential diagnoses do you think about? What do you think of the methodologies for diagnosing of children with bilateral edema? What information is collected before making the diagnosis (anamnesis, etc.)? How do you link bilateral edema to Kwashiorkor? What conditions and diseases are considered in the differential diagnosis of Kwashiorkor or other conditions related to edema? What reasons for seeking care are given by families when they bring children with edema? Do families have any knowledge, in your opinion, of the cause of the child's condition?

4. *Treatments for patients:*

Do you think that are inconsistencies between diagnosis and speed of recovery? If so, why? How do you think we could improve their state of health and reduce mortality in this population? Do you think we should involve more the communities? If so, which efforts can we make to better involve communities?

*5. Final thoughts of the participant (if appropriate).*
