# Supplementary material for: Understanding the medical challenges for the diagnosis and treatment of bilateral pitting oedema in children: a qualitative study
Source: PLOS Glob Public Health. 2025 Mar 18;5(3):e0004125. doi: 10.1371/journal.pgph.0004125 (PMC11918406; doi:10.1371/journal.pgph.0004125)
Supplement: S2 Text — (DOCX) [file pgph.0004125.s002.docx]

S2 Text: Focus group discussion guide

This interview will not be used as an evaluation of your work.This interview will not be used as an evaluation of your work. We want only to know your opinion and perceptions on the process of diagnosing the causes of bilateral edema.

1. *Diagnosis of pathologies presenting with edemas*

Who usually diagnoses pathologies presenting with bilateral edema? What is the role of nurses and doctors? What about other professionals (nutritionist, nephrologist, etc.)? Is there collaboration between doctors, nurses and other health professionals?

What difficulties do you encounter in the process of diagnosing children with bilateral edema? In determining the reasons behind edema? What do you think of the training of health professionals in the diagnosis and care of children with bilateral edema? How can we improve care in your opinion?

What is needed to better understand the current method of diagnosing children with bilateral edema? Is the training of health professionals working with children with nutritional edema adequate? What kind of additional or different training would be helpful for medical staff involved in working with children with nutritional edema?

*2.Treatment:*

How is the treatment of bilateral edema carried out? What does it depend on? What do you think about it? If the child is malnourished, is the differential diagnosis and treatment done in conjunction with the nutrition treatment or is it done differently?

3.*Final thoughts (if appropriate)*
